# Supplementary material for: Nucleation and strain-stabilization during organic semiconductor thin film deposition
Source: Sci Rep. 2016 Sep 7;6:32620. doi: 10.1038/srep32620 (PMC5013491; doi:10.1038/srep32620)
Supplement: Supplementary Information [file srep32620-s1.pdf]

Nucleation and strain-stabilization during organic semiconductor thin film deposition

Yang Li,[1] Jing Wan,[1] Detlef-M. Smilgies,[2] Nicole Bouffard,[3] Richard Sun,[4] and Randall L. Headrick[1, a)]

[1)]Department of Physics and Materials Science Program, University of Vermont, Burlington VT 05405

[2)]Cornell High Energy Synchrotron Source, Cornell University, Ithaca NY 14853

[3)]Microscopy Imaging Center, College of Medicine, University of Vermont, Burlington VT 05405

[4)]Angstrom Sun Technologies Inc. 31 Nagog Park, Acton MA 01720

[a)]Email: rheadrick@uvm.edu

Supplementary information

Contents:

1) Supplementary results for Section: In-situ optical monitoring of the crystallization process.

    a. Optical reflectance peak positions: Table S1.

    b. Absorption spectrum: Fig. S1.

    c. Real-time X-ray scattering study: Fig. S2.

2) Supplementary results for Section: Strain-free optical spectra vs. deposition temperature.

    a. Film thickness versus deposition temperature: Fig. S3.

    b. Additional reflectance spectra and peak positions: Fig. S4.

3) Supplementary results for Section: Metastable polymorph fabrication and stabilization.

    a. Optical microscope and AFM images of thin films prepared with isotropic grain structures: Figs. S5, S6 and S7.

    b. GIWAXS of Form I sample (Fig. S8) and in-plane peak positions for both Form I and Form II samples (Table S2).

1) Supplementary results for Section: In-situ optical monitoring of the crystallization process.

a. Optical reflectance peak positions.

Table S1. Reflectance features of TIPS-pentacene (8.7 mg/ml in toluene, 25 ˚C, 0.4 mm/s); silicon wafers were used as substrates, Ten reflection spectra have been collected for each region and the standard deviation of each peak position was calculated. Uncertainties for peak shifts include a systematic uncertainty of 6-7 nm due to the possiblility of dispersive effects in reflection mode measurements. Typical reflection spectra for each region can be seen in Fig. 2 in the main text.

	Intramolecular absorption wavelength	$S_0 \rightarrow S_1$ absorption wavelength		
	(nm)	0→2 (nm)	0→1 (nm)	0-0 (nm)
In solution at meniscus (liq.)	439(1)	550(1)	593(1)	644(1)
Supersaturated region (s.s.)	441(2)	555(2)	604(3)	659(3)
Solid film	442(2)	583(2)	644(3)	700(3)
Peak shift: liq. to s.s.	2(2)	5(2)	11(3)	15(3)
Peak shifts: liq. to solid	5(2)	33(2)	51(3)	56(3)

b. Absorption spectrum.

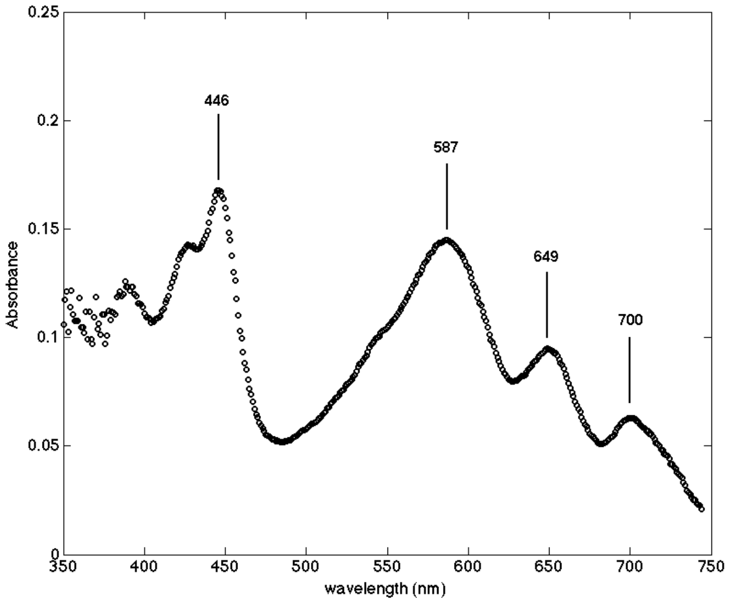

Fig. S1. Absorption spectrum for a 82 nm thin film of TIPS-Pentacene on UV-ozone treated glass (deposited from a 1.5 mg/ml solution with toluene as the solvent; substrate temperature: 25 ˚C; writing speed: 0.05 mm/s).

c. Real-time X-ray scattering study.

In-situ μGIWAXS is carried out to study TIPS-pentacene thin film crystallization. The capillary is moving for this experiment rather than the substrate. The writing speed is 0.4 mm/s at 25˚C and the concentration is 8.7 mg/ml. As the capillary is moving, the capillary passes the area illuminated by the X-ray beam, followed by meniscus, supersaturated region and finally the solid film, producing a real-time intensity record with a time resolution of 0.1 s, and an effective spatial resolution of ~100 μm. Note that the effective spatial resolution is mainly limited by a small curvature of the contact line where the meniscus meets the substrate surface coupled with the fact that the X-ray footprint is elongated along the contact line due to the grazing incidence of the X-rays. The in-situ X-ray results are shown in Fig. S1. We set the time at which the capillary passes the X-ray beam to be $t = 0$ s. The change of the integrated Bragg peak intensities versus time is plotted in Fig. S1(a). At $t = 0$ s, a broad ring from toluene scattering appears, which disappears again at $t = 1.3$ s. This marks the point at which the X-ray beam illuminates the supersaturated region. We also observe that at $t = 1.3$ s, the measured (001) peak intensity is only 16% of its final value. We believe that the supersaturated region does not contribute significantly to this intensity and we attribute the small measured intensity to the limited spatial and temporal resolution in the experiment – that is, a small part of the X-ray beam is already illuminates the crystalline part of the film at this moment. Subsequently, after the initial rapid intensity increase, the (001) and (101) intensities continue to increase at a slower rate for more than 10 s. This indicates that the ordering of the crystalline film continues to improve.

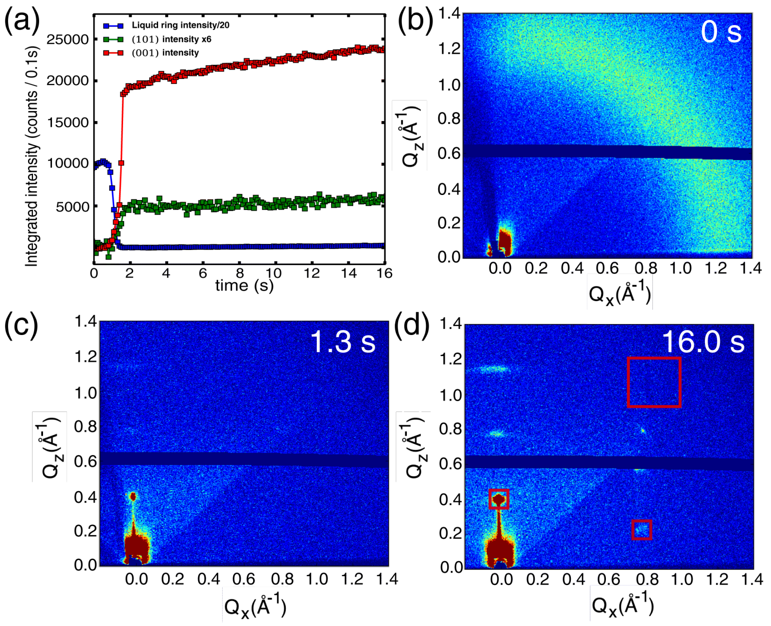

Fig. S2. (a) Integrated Bragg peak intensities versus time. The capillary passes the X-ray beam at $t = 0$ s. (b) The x-ray scattering pattern at $t = 0$ s. (c) the diffraction pattern at $t = 1.3$ s when toluene ring has just disappeared. (d) the final diffraction pattern. The red boxes in (d) show the regions used to calculate peak intensities for (001), (101) and the liquid ring.

2) Supplementary results for Section: Strain-free optical spectra vs. deposition temperature.

a. Film thickness versus deposition temperature.

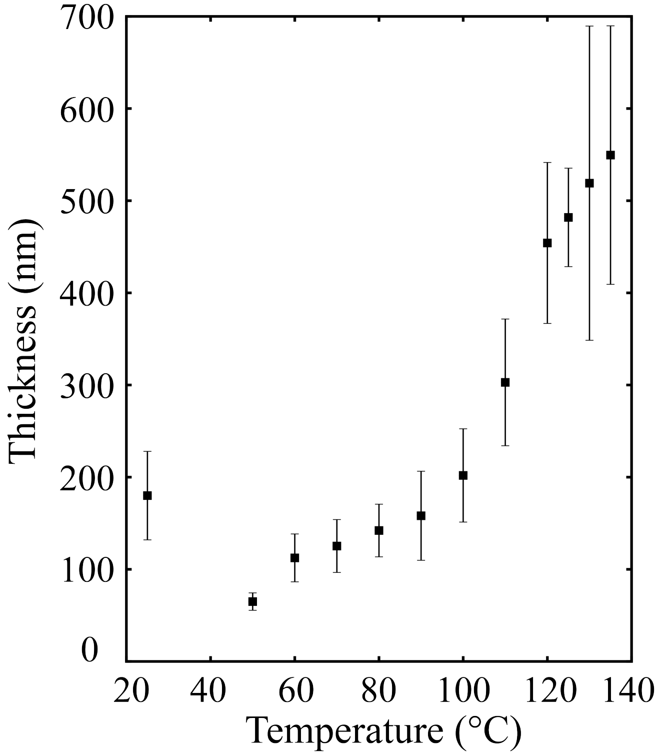

Fig. S3. TIPS-Pentacene film thickness as a function of deposition temperature. The depositon conditions for 50 – 135 °C are: 1.5 mg/ml in mesitylene at 0.05 mm/s, and for 25 °C are: 1.5 mg/ml in toluene at 0.05 mm/s. UV-ozone treated glass slides were used as the substrates for all of the samples. Uncertainties are dominated by variations of the film thickness within individual samples. Film thickness was measured with a Bruker DektakXT profilometer.

b. Additional reflectance spectra and peak positions

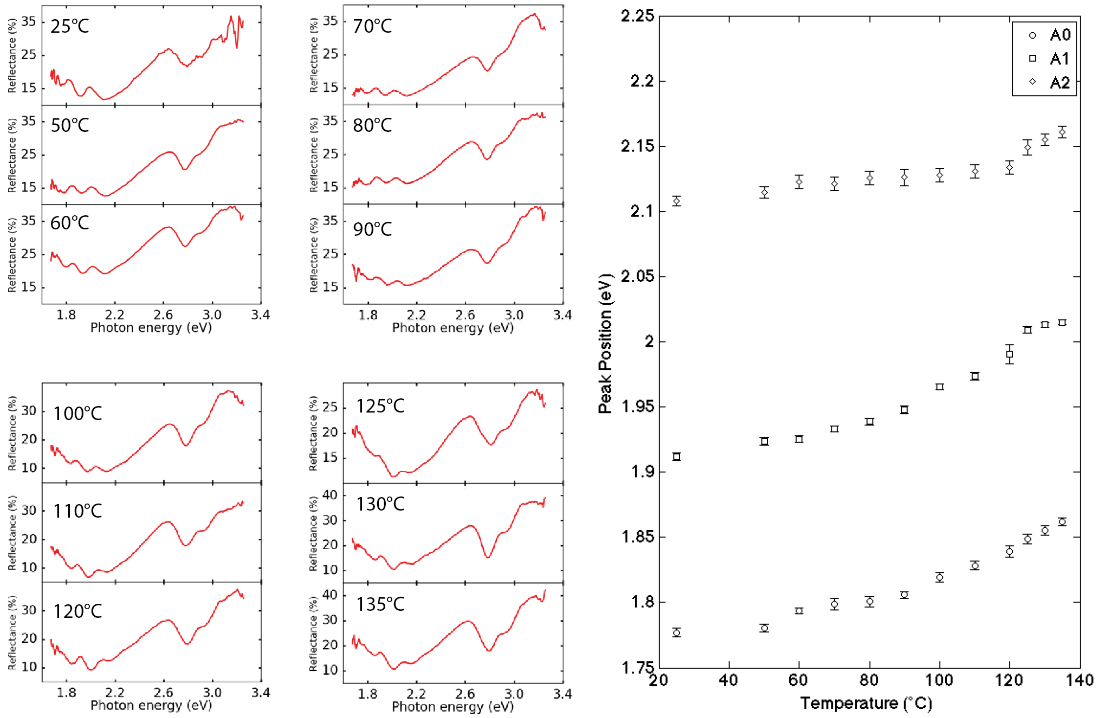

Fig. S4. Examples of reflectance spectra for every temperature shown in Fig. 4(b) of the main text. The reflection spectra were measured at the deposition temperature for each sample, as described in the main text. Peak position versus temperature for three reflection peaks (A_0, A_1, A_2) are also shown. Mean peak positions and standard deviations are derived from five measurements in different spots on each sample.

3) Supplementary results for Section: Metastable polymorph fabrication and stabilization.

a. Optical microscope and AFM images of thin films prepared with isotropic grain structures.

The images below in Figs. S5-S7 show that the as-deposited grain structure for samples A, B, and D are still intact after the heating and cooling cycles that each sample was subjected to.

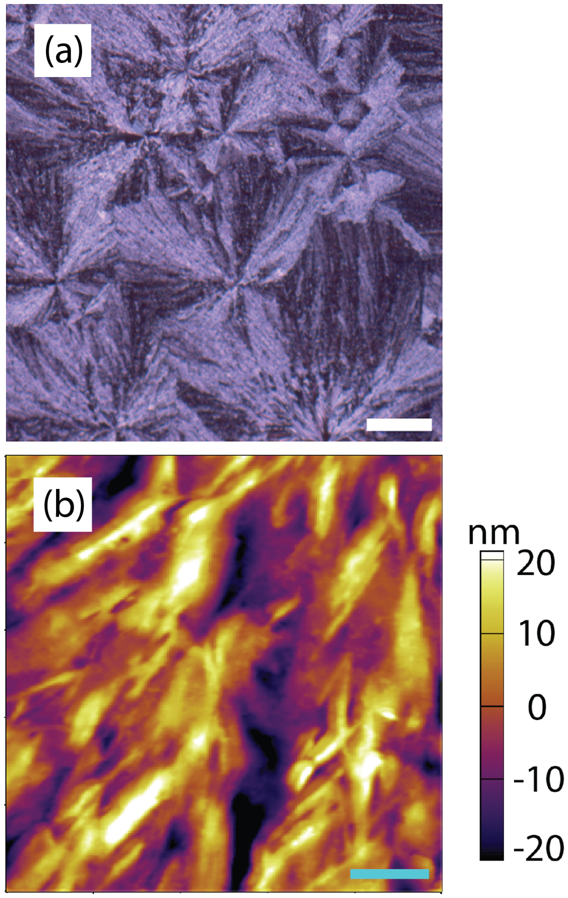

Fig. S5. Optical (a) and AFM (b) images of Form I after the heating and cooling cycle shown in Fig. 7(a) of the main text. The sample is listed as sample A in Table II of the main text. After deposition, this film was heated up to 135˚C and then cooled down to 25˚C before these images were recorded. The scale bar in (a) is 50 μm and in (b) is1 μm.

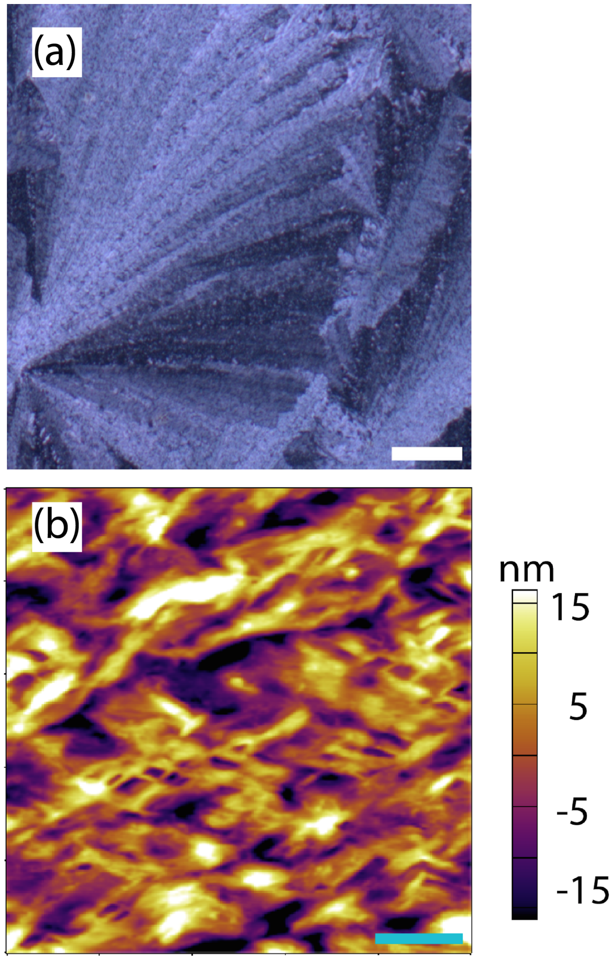

Fig. S6. Optical (a) and AFM (b) images of a Form II thin film after cooling and heating cycle shown in Fig. 7(a) of the main text. The sample is listed as sample B in Table II of the main text. The sample was cooled down to 25˚C, heated up to 135˚C and then cooled down to 25˚C again before these images were recorded. The scale bars in (a) and (b) are 50 μm and 1 μm respectively.

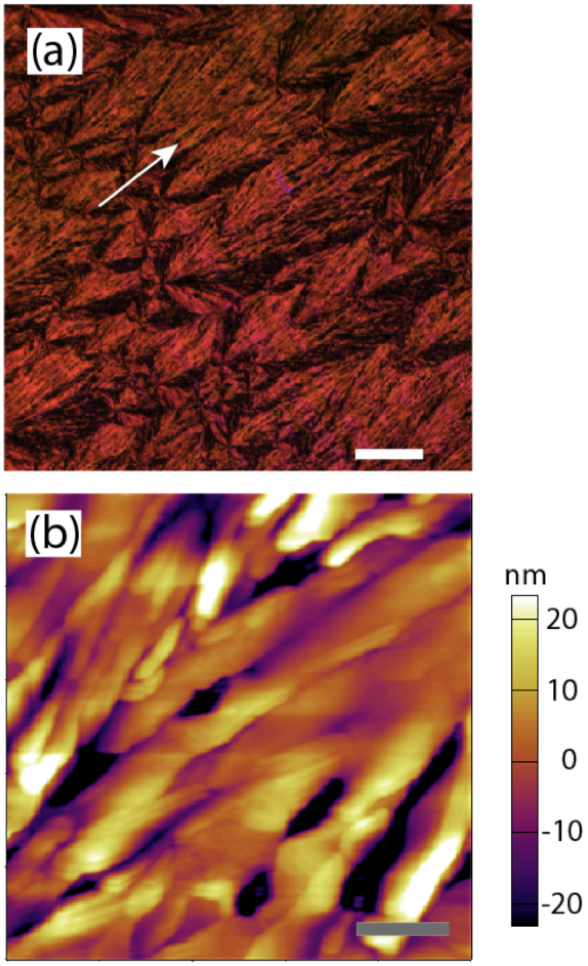

Fig. S7. Optical microscopy and AFM images of the Form II sample referred to as D in Tables II and III of the main text. The GIWAXS pattern for this sample is shown in Fig. 7(b) of the main text. The arrow in the optical image shows the motion stage moving direction. The scale bar of the microscopy image is 200 μm and the scale bar of AFM image is 1 μm.

b. GIWAXS of Form I sample and in-plane peak positions for Form I and II.

Sample C is a reference sample prepared at 25°C, and was not subjected to thermal cycling.

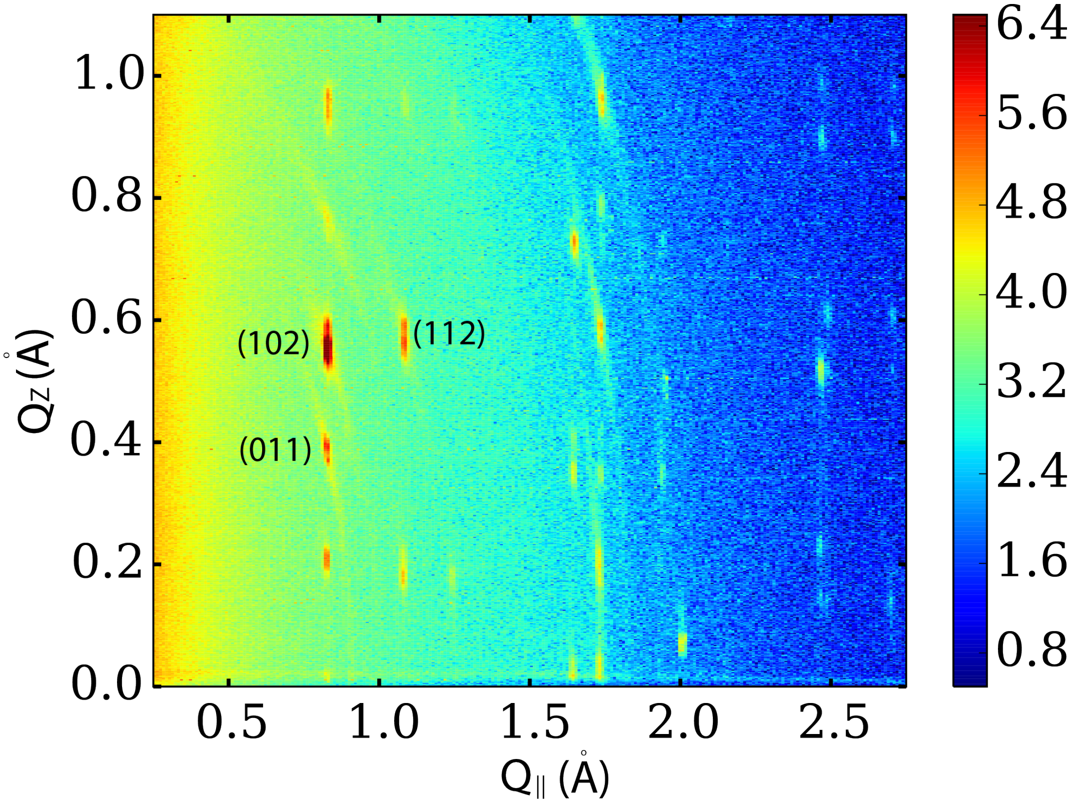

Fig. S8. X-ray scattering of Form I TIPS-pentacene. The film was made at 25°C on a Si/SiO_2 substrate. This sample is referred to as sample C in Tables II and III of the main text. For comparison, the X-ray scattering of Form II is shown in Fig. 10 of the main text.

Table S2. Summary of $Q_{||}$ values of the major Bragg peaks present for the different polymorphs. $Q_{||}$ values of Form I are obtained from Fig. S8. $Q_{||}$ values of Form II are obtained from Fig. 10 in the paper. The $Q_{||}$ of (10L), (01L) and (11L) for both thin film forms were calculated by choosing (102), (011) and (112) peaks. The values are used to calculate the *a*, *b* and γ lattice constants, which are listed in Table III in the main text.

Phase	$Q_{\|\|}$ ($Å^{-1}$) (10L)	$Q_{\|\|}$ ($Å^{-1}$) (01L)	$Q_{\|\|}$ ($Å^{-1}$) (11L)
Form I made at 25°C	0.825	0.821	1.06
Form II made at 135°C	0.755	0.864	0.937
Form II cooled to 25°C	0.762	0.861	0.946
